# Supplementary material for: Identification of photoperiod-induced specific miRNAs in the adrenal glands of Sunite sheep (Ovis aries)
Source: Front Vet Sci. 2022 Jul 22;9:888207. doi: 10.3389/fvets.2022.888207 (PMC9354845; doi:10.3389/fvets.2022.888207)
Supplement: Supplementary file 1 [file Data_Sheet_1.ZIP › Supplementary file/miRDeep2 file.zip!/miRDeep2 file/Know_Structure/LP42A3/oar-mir-487a.pdf]

miRBase precursor : oar-mir-487a
Total read count : 225
oar-miR-487a-5p read count : 21
oar-miR-487a-3p read count : 201
remaining reads : 3

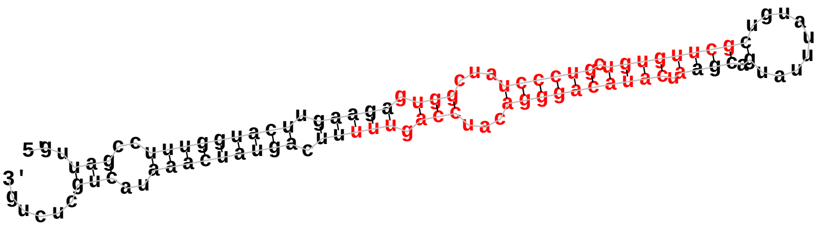

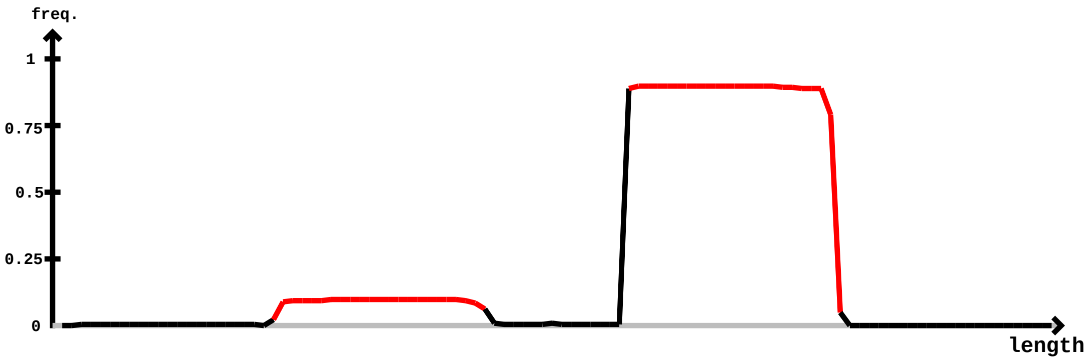


```
                                                                    oar-miR-487a-3p
                          oar-miR-487a-5p
5'- guuagccuuugguacuugaagaguggcuaucccugcuguguucgcuguauuuaugacgaaucauacagggacauccaguuuuucaguaucaaauacugcucug   -3'   exp
    ..(((..(((((((((.(((((.(((...((((((.(((((((((.........).)))).))))))))))...))).))))).)))))))))..))).....     reads   mm   sample
    .uuagccuuugguacuugaa...................................................................................     1       0    seq
    .....................aguggcuaucccugcuguguu.............................................................     2       0    seq
    .....................aguggcuaucccugcuguguuc............................................................     2       0    seq
    .....................aguggcuaucccugcuguguucg...........................................................     1       0    seq
    ......................guggcuaucccugcugugu..............................................................     1       0    seq
    ......................guggcuaucccugcuguguuc............................................................     2       0    seq
    ......................guggcuaucccugcuguguucg...........................................................     11      0    seq
    ......................guggcuaucccugcuguguucgc..........................................................     1       0    seq
    .......................uggcuaucccugcuguguuc............................................................     1       0    seq
    ...........................uaucccugcuguguucgcuguauu....................................................     1       0    seq
    ..................................................uuaugacgaaucauacagggacau.............................     1       0    seq
    ...........................................................aucauacagggacaucc...........................     1       0    seq
    ...........................................................aucauacagggacauccagu........................     22      0    seq
    ...........................................................aucauacagggacauccaguu.......................     166     0    seq
    ...........................................................aucauacagggacauccaguuu......................     10      0    seq
    ............................................................aucauacagggacauccaguu......................     1       0    seq
    ............................................................aucauacagggacauccaguuu.....................     1       0    seq
```
